# The complete chloroplast genome sequence and phylogenetic analysis of *Hordeum pusillum* Nutt., 1818 (Poaceae)

**DOI:** 10.1080/23802359.2024.2412225

**Published:** 2024-10-07

**Authors:** Qingwei Du, Suping Yu

**Affiliations:** a Beijing Key Laboratory of Agricultural Genetic Resources and Biotechnology, Institute of Biotechnology, Beijing Academy of Agriculture and Forestry Sciences, Beijing, China; b School of Ecology and Environment, Beijing Technology and Business University, Beijing, China; c State Environmental Protection Key Laboratory of Food Chain Pollution Control, Beijing Technology and Business University, Beijing, China; d Key Laboratory of Cleaner Production and Integrated Resource Utilization of China National Light Industry, Beijing Technology and Business University, Beijing, China

**Keywords:** Chloroplast genome, *Hordeum pusillum*, Poaceae, phylogenetic analysis

## Abstract

*Hordeum pusillum* (*Hordeum pusillum* Nutt., 1818) is an annual barley that is native to the west of the North America and widespread in southern United States and tropical America. In this study, we have provided the first complete characterization of the chloroplast genome of *H. pusillum*. Our research revealed that the circular chloroplast genome of *H. pusillum* consists of a large single-copy region (LSC: 80,853 bp), a small single-copy region (SSC: 12,745 bp), and a pair of inverted repeat regions (IRs: 21,587 bp), totaling 136,772 bp in length. Within the chloroplast genome of *H. pusillum*, 91 protein-coding genes, 38 tRNA genes, and ten rRNA genes were identified. To determine the evolutionary relationship of *Hordeum* species with reported chloroplast genome sequences, we constructed a phylogenetic tree using the entire chloroplast genome sequences. The evolutionary position of *H. pusillum* corresponds to its geographical location. The chloroplast genome of *H. pusillum* provided in this study may have significant implications for the phylogenetic study of Poaceae species.

## Introduction

*Hordeum pusillum* Nutt., also known as little barley grass, is a native food that has been domesticated in the southwest of the United States (Graham et al. 2017). It was considered a significant ancient food as it provided a source of food during a typically scarce time of year in late spring. Based on the genotype of the genome, *Hordeum* can be classified into four types: I, Xa, Xu, and H genomes (Blattner 2009). The *Hordeum* I genome can be further divided into Old World and New World species based on their geographical location. *H. pusillum* is a representative species of the I genome in the New World. Currently, there has not been a complete characterization of the chloroplast genome of *H. pusillum* reported, and the evolutionary relationship of *Hordeum* species based on chloroplast genome sequences remain unclear. Therefore, this study aims to elucidate the relationships among closely related *Hordeum* species. In this study, we sequenced and assembled the complete chloroplast genome of *Hordeum pusillum* Nutt. This chloroplast genome will promote taxonomic studies and the development of molecular markers for *Hordeum* species.

## Materials and methods

### Plant sampling

**Figure 1.**
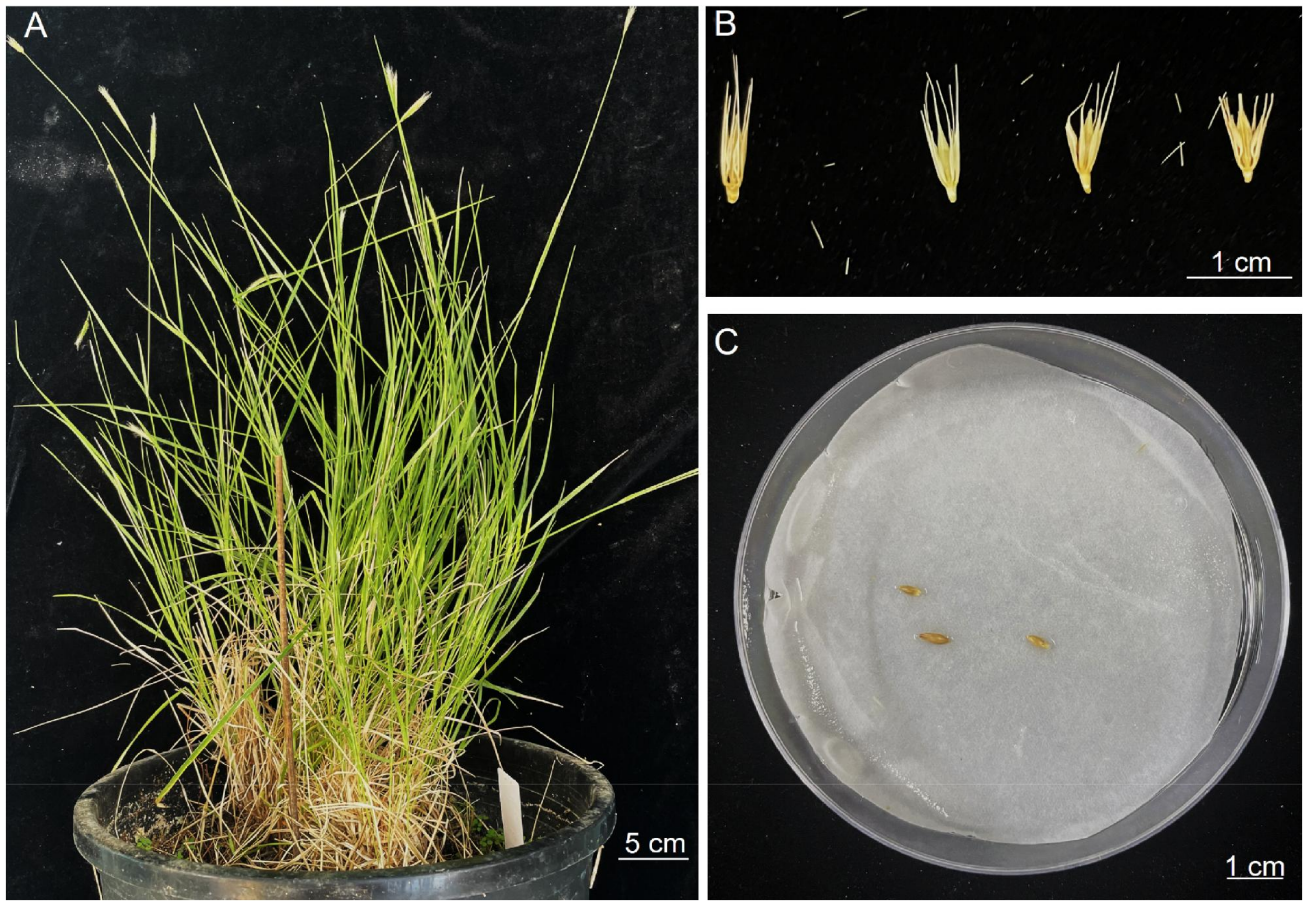
Species reference image of *H. pusillum* Nutt. (voucher no. BTBU20230513) from our laboratory were photographed by Suping Yu. (A) Seedlings of *H. pusillum* Nutt. under cultivation. Stems can be singular or multiple originating from the base, with leaf blade width ranging from 1.5 to 6 mm. (B) Seeds of *H. pusillum* Nutt. Florets are surrounded by a pair of bracts called the lemma and palea. (C) Hydroponic germination of *H. pusillum* Nutt. seeds, which are oval-elliptic and smooth.

The seeds of *H. pusillum* were obtained from The National Small Grains Collection (NSGC) of the United States (west latitude 76°8′47.391″, north longitude 39°30′58.477″). *H. pusillum* has been cultivated in our laboratory, as illustrated in Figure 1. Seedling leaves were applied to extract total genomic DNA using a Plant Genomic DNA Kit (Tiangen Biotech, Beijing, China). The specimen has been deposited in the School of Ecology and Environment (Beijing Technology and Business University https://www.btbu.edu.cn/, contact person: Suping Yu, yu.su.ping@163.com) under the voucher number BTBU20230513.

### Sequencing, assembly, and annotation

DNA Library construction and sequencing were performed on HiSeq 4000 platform with 150-bp paired-end mode (Novogene). FastQC v0.12.1 was applied for quality control of raw sequencing reads (Andrews 2010). Low-quality reads were filtered out by Trimmomatic v0.40 (Bolger et al. 2014). The clean reads were used to assemble the complete chloroplast genome by GetOrganelle v1.1.7 (Jin et al. 2020). Sequencing depth was evaluated by BWA v0.7.17 (Li 2013) and samtools v1.16.1 (Li et al. 2009). The chloroplast genome was annotated by GeSeq (https://chlorobox.mpimp-golm.mpg.de/geseq.html) (Tillich et al. 2017) and visualized using Chloroplot (Zheng et al. 2020).

### Phylogeny analysis

The sequences of chloroplast genomes were downloaded from NCBI (http://www.ncbi.nlm.nih.gov/). A multiple-sequence alignment of the complete chloroplast genome sequences of 16 species including 7 *Hordeum* species was performed using MAFFT (Katoh et al. 2002). The best-fit nucleotide substitution model ‘GTRGAMMAX’ was selected by ModelTest-NG v 0.1.6 (Darriba et al. 2020). Maximum-likelihood (ML) evolutionary trees were constructed using RAxML-NG (Kozlov et al. 2019) with 1000 bootstrap replicates. The phylogenetic tree was visualized by online iTOL (https://iTOL.embl.de/).

## Results

**Figure 2.**
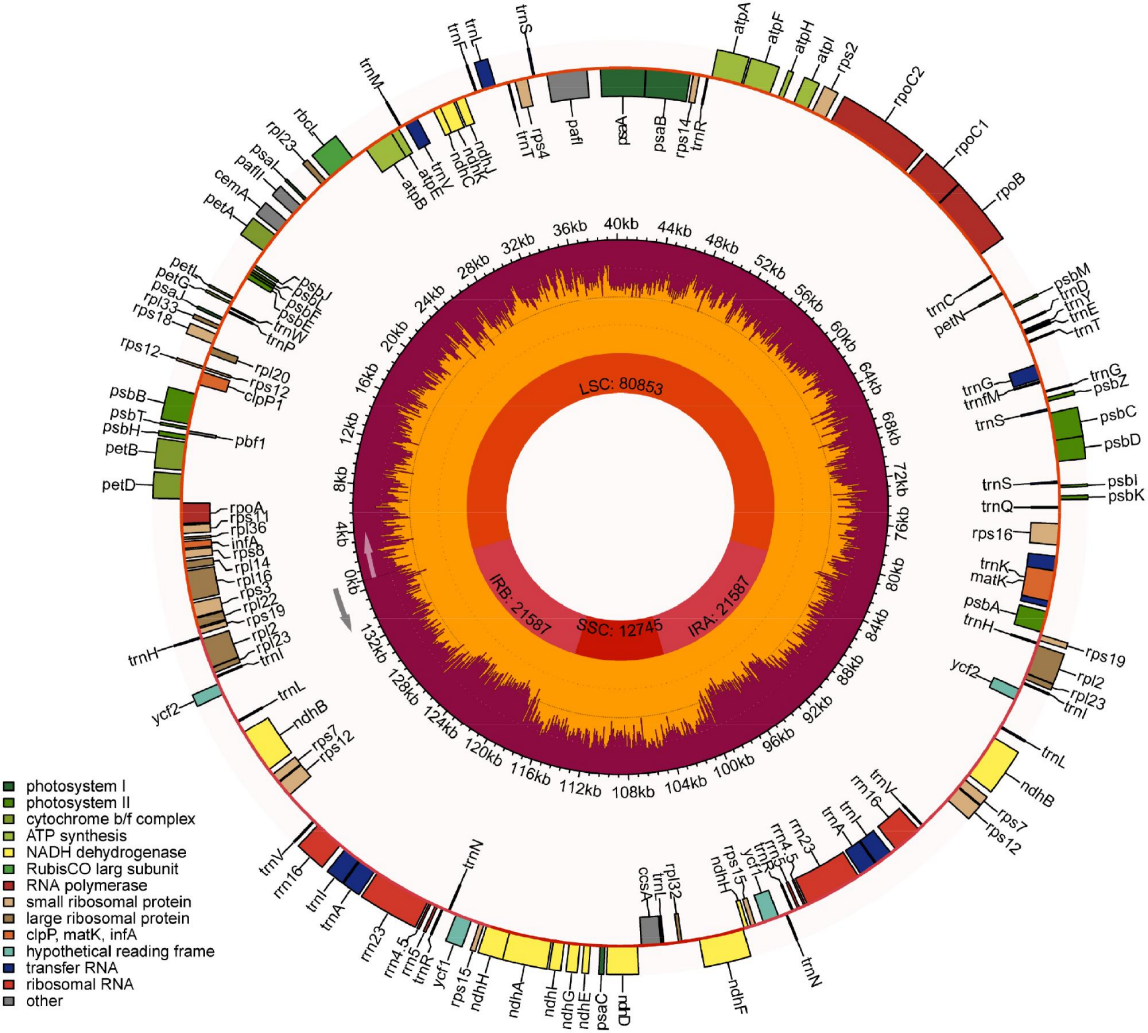
Circular chloroplast genome structure map of *H. pusillum*. The genetic map includes three circles: the inner circle displays the locations of the LSC, SSC, IRA, and IRB regions. The Middle circle represents the corresponding GC content. The outer circle indicates genes with various functions, and gene categories are displayed in different colors in the lower left corner.

**Figure 3.**
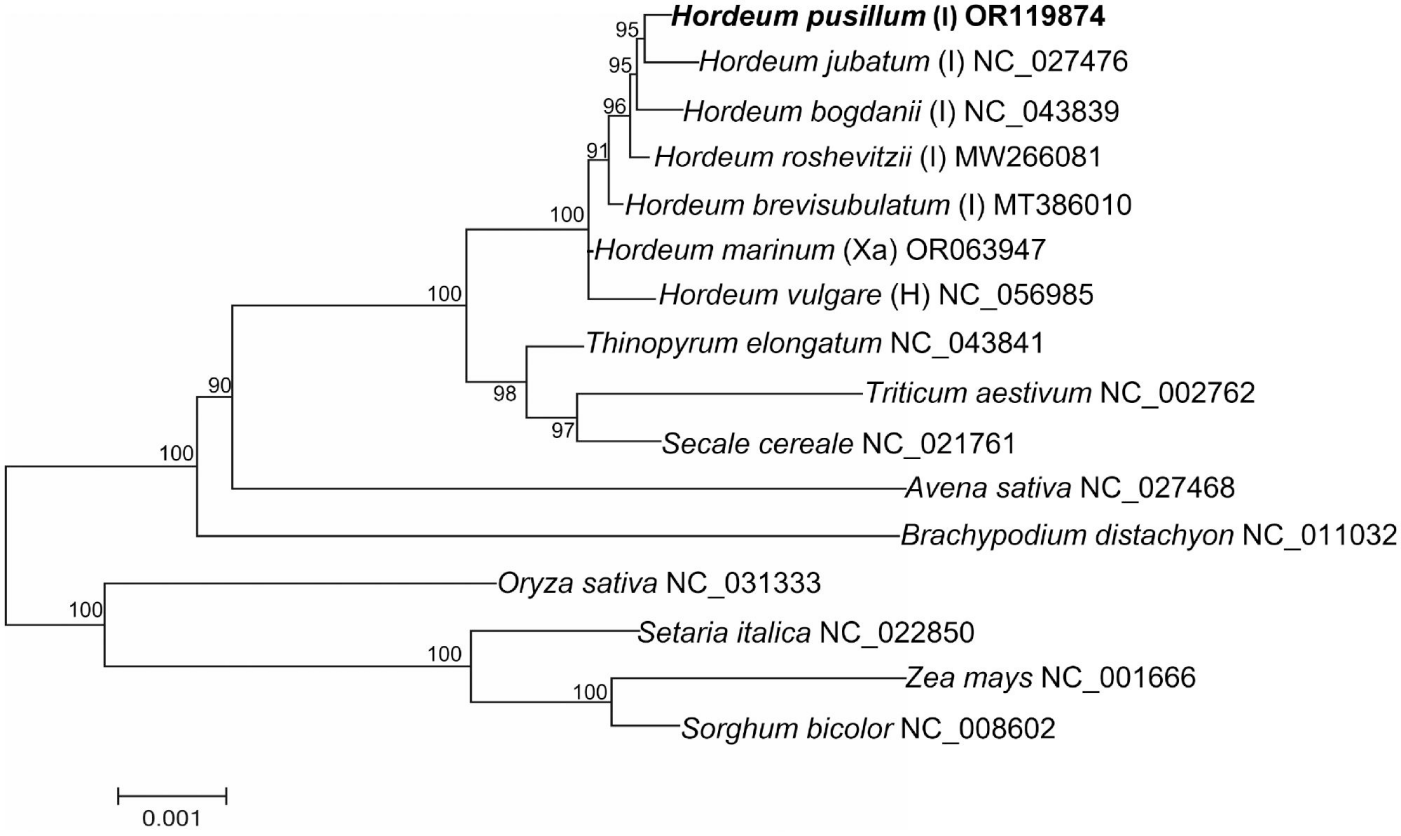
The ML phylogenetic tree is based on whole chloroplast genome sequences of 16 species. The complete chloroplast genomes of 15 species, including *Hordeum jubatum* (NC_027476), *Hordeum bogdanii* (NC_043839), *Hordeum roshevitzii* (MW266081), *Hordeum brevisubulatum* (MT386010) (Su et al. 2020), *Hordeum marinum* (OR063947) (Yu and Du 2023), *Hordeum vulgare* (NC_056985), *Thinopyrum elongatum* (NC_043841), *Triticum aestivum* (NC_002762) (Ogihara et al. 2002), *Secale cereale* (NC_021761) (Middleton et al. 2014), *Avena sativa* (NC_027468) (Saarela et al. 2015), *Brachypodium distachyon* (NC_011032) (Bortiri et al. 2008), *Oryza sativa* (NC_031333), *Setaria italica* (NC_022850), *Zea mays* (NC_001666) (Maier et al. 1995) and *Sorghum bicolor* (NC_008602) (Saski et al. 2007) were downloaded from NCBI (www.ncbi.nlm.nih.gov/).

The complete chloroplast genome of *H. pusillum* was assembled, with a length of 136,772 bp. The overall GC content of the chloroplast genome was 38%, and the average coverage depth of the chloroplast genome assembly was 4982× (Figure S1). The chloroplast genome structure of *H. pusillum* included a small single-copy region (SSC: 12,745 bp), a large single-copy region (LSC: 80,853 bp), and a pair of inverted repeat regions (IRs: 21,587 bp) (Figure 2). A total of 137 genes were annotated from the chloroplast genome, including 91 protein-coding genes, 38 tRNA genes and 8 rRNA genes. 20 genes were identified as containing introns, with *atpF* and *ndhA* each having two introns. Additionally, *atpF*, *ndhA*, *ndhB*, *pafI*, *petB*, *petD*, *rpl16,* and *rps12* were identified as being difficult to annotate (Figure S2). Our phylogenetic tree, constructed using 16 whole chloroplast genome sequences, revealed the evolutionary relationships of species in the Poaceae family (Figure 3). The relationship of *Hordeum* species is generally consistent with previous reports (Blattner 2009), the New World species (*H. pusillum* and *H. jubatum*) and the Old World species (*H. bogdanii*, *H. roshevitzii* and *H. brevisubulatum*) of I genomes clustered into one branch. In addition, Xa genome species (*H. marinum*) and H genome species (*H. vulgare*) clustered sequentially outside the I genome branch.

## Discussion and conclusion

The *Hordeum* genus is a medium-sized genus in the Triticeae tribe of the Poaceae family. *Hordeum* species contain valuable traits for resisting pathogens and adapting to harsh environmental conditions, which could greatly benefit other cereals if successfully transferred. In recent years, *H. pusillum* has gained more attention due to its unique evolutionary position and remarkable native food value. However, the complete chloroplast genome sequences of *H. pusillum* and the evolutionary relationship of *Hordeum* species based on whole chloroplast sequences are still not well understood. This study supplements and improves upon previous phylogenetic analysis results of *H. pusillum* (Blattner 2009), indicating the important role of chloroplast genomes as molecular markers. It reveals that the chloroplast genome sequences can effectively distinguish the evolutionary relationship of three genomic species (I, Xa and H) in the *Hordeum* genus. The finding suggests that geographic location and environment could have driven the convergent evolution of Xa genome species and I genome species. Therefore, the complete chloroplast genome of *H. pusillum* provides a valuable genetic resource for future research on the taxonomy and evolution of the Poaceae family and *Hordeum* genus.

## Data Availability

The chloroplast genome data supporting the findings of this study have been publicly available in NCBI. The complete chloroplast genome sequence of *H. pusillum* was submitted to GenBank with the accession number OR119874.1. The associated BioProject is PRJNA1111310; the SRA number is SRR29044702; and the Bio-Sample number is SAMN41387973, respectively.
